# Nicorandil Regulates Ferroptosis and Mitigates Septic Cardiomyopathy *via* TLR4/SLC7A11 Signaling Pathway

**DOI:** 10.1007/s10753-023-01954-8

**Published:** 2023-12-30

**Authors:** Jin-shuai Lu, Jian-hao Wang, Kun Han, Nan Li

**Affiliations:** 1 Departments of Emergency, People’s Hospital of Xinjiang Uygur Autonomous Region, Urumqi City, Xinjiang 830001 China; 2 Postgraduate School, Xinjiang Medical University, Urumqi City, Xinjiang 830017 China; 3 Xinjiang Emergency Center, People’s Hospital of Xinjiang Uygur Autonomous Region, Urumqi City, Xinjiang 830001 China

**Keywords:** septic cardiomyopathy, nicorandil, ferroptosis, toll-like receptor (TLR) 4/solute carrier (SLC) 7A11 signaling pathway.

## Abstract

This study mainly explored the role of nicorandil in regulating ferroptosis and alleviating septic cardiomyopathy through toll-like receptor (TLR) 4/solute carrier family 7 member 11 (SLC7A11) signaling pathway. Twenty-four male SD rats were randomly divided into control, Nic (nicorandil), LPS (lipopolysaccharide), and LPS + Nic groups and given echocardiography. A detection kit was applied to measure the levels of lactic dehydrogenase (LDH), cardiac troponin I (cTnI), and creatine kinase-MB (CK-MB); HE staining and the levels of glutathione (GSH), malondialdehyde (MDA), total iron, and Fe^2+^ of myocardial tissues were detected. Moreover, the expression of TLR4 and SLC7A11 were measured by qRT-PCR and the proteins regulating ferroptosis (TLR4, SLC7A11, GPX4, ACSL4, DMT1, Fpn, and TfR1) were checked by western blot. Myocardial cells (H9C2) were induced with lipopolysaccharide (LPS) and transfected with si-TLR4 or SLC7A11-OE. Then, the viability, ferroptosis, and TLR4/SLC7A11 signaling pathway of cells were examined. Nicorandil could significantly increase left ventricular (LV) ejection fraction (LVEF) while reduce LV end-diastolic volume (LVEDV) and LV end-systolic volume (LVESV). Also, it greatly reduced the levels of LDH, cTnI, and CK-MB; alleviated the pathological changes of myocardial injury; notably decreased MDA, total iron, and Fe^2+^ levels in myocardial tissues; and significantly increased GSH level. Besides, nicorandil obviously raised protein levels of GPX4, Fpn, and SLC7A11, and decreased protein levels of ACSL4, DMT1, TfR1, and TLR4. After knockdown of TLR4 or overexpression of SLC7A11, the inhibition effect of nicorandil on ferroptosis was strengthened in LPS-induced H9C2 cells. Therefore, nicorandil may regulate ferroptosis through TLR4/SLC7A11 signaling, thereby alleviating septic cardiomyopathy.

## Introduction

Sepsis, one of the leading causes of death in critically ill patients, is a complex disease process wherein the body’s response to a pathogen is amplified far beyond the initial site of infection. Multiple organ dysfunction syndrome, a characteristic outcome of severe sepsis, is caused by tissue hypoperfusion as a result of hemodynamic changes. The heart is an important target organ for severe sepsis/septic shock. Septic cardiomyopathy is a serious complication of sepsis and is directly associated with a high mortality rate in sepsis [1]. Cardiac dysfunction is a critical manifestation of septic patients. About 60% of septic patients admitted to intensive care unit have clinical manifestations of cardiac dysfunction, and 70–90% of them die. As for septic patients without cardiac involvement, their mortality rate is only 20% [2]. Recently, with the improvement of medical technology, great progress has been made in the study of septic cardiomyopathy in terms of molecular biology or clinical diagnosis and treatment. Some researchers have continuously proposed the related pathological mechanism of septic cardiomyopathy [3, 4]. Septic cardiomyopathy is still a serious complication of sepsis with a high mortality rate. It is reported that conventional therapy with chemotherapeutic agents (positive inotropes, beta-1-blockers, melatonin, and dexmedetomidine) and traditional Chinese medicine (TCM) are two main alternatives for the treatment of septic cardiomyopathy [5–11].

Nicorandil is an adenosinetriphosphate sensitive K (K-ATP) channel opener and a nitric oxide donor. Coronary artery or intravenous injection of nicorandil has been widely recognized and applied in the treatment of acute myocardial infarction, acute heart failure, arrhythmia, and other conditions in clinical practice [12]. Recent studies have confirmed that nicorandil has the effects of anti-inflammation, anti-oxidative stress, anti-apoptosis, and stabilizing and restoring mitochondria. Nicorandil also has a good prospect for the protection of visceral organs, especially the heart [13]. Moreover, nicorandil significantly improves cardiac insufficiency by inhibiting toll-like receptor (TLR) 4/solute carrier (SLC) 7A11 signaling pathway and blocking GSDMD activation. By reducing the release of pro-inflammatory cytokines, nicorandil can significantly inhibit the occurrence of ferroptosis [14]. Some studies have pointed out that nicorandil can reduce the release of inflammatory factors in septic rats, improve the inflammatory response, relieve myocardial damage, and protect myocardium [15]. However, the specific mechanism of nicorandil on septic cardiomyopathy is still unclear.

Ferroptosis is a new type of regulated cell death that is dependent on reactive oxygen species (ROS) and iron. Some studies indicate an important role of ferroptosis in myocardial injury caused by sepsis [16]. TLR4 is a crucial regulator of apoptosis and autophagy, and its upregulation may be correlated with ferroptosis [17]. The cytoplasm of TLR4 is also known as the toll/interleukin-1 receptor (TIR) domain. The TLR4 can transmit lipopolysaccharide (LPS)-mediated signals to cells through a variety of proteins, activate nuclear factor-κB (NF-κB) and c-Jun N-terminal kinase (JNK)/p38 signaling pathways, and release downstream inflammatory factors such as tumor necrosis factor (TNF)-α, interleukin (IL)-6, IL-8, and IL-1 β. Collectively, TLR4 is able to amplify the inflammatory cascade at the early stage of sepsis [18]. Solute carrier family 7 member 11 (SLC7A11), a member of SLC family, can promote the synthesis of glutathione (GSH), and inhibiting its expression can induce ferroptosis [19]. Moreover, TLR4 has been revealed to induce ferroptosis through SLC7A11-mediated signal pathway [20]. Several studies demonstrated that nicorandil reduced myocardial injury by inhibiting TLR4 signal transduction [14, 21]. However, no study has been performed to explore the relationship between nicorandil and ferroptosis. Therefore, to make up for research gaps, the mechanism through which nicorandil treats septic cardiomyopathy *via* the TLR4/SLC7A11 signaling was investigated in this study. We expected to alleviate the myocardial injury in patients with septic cardiomyopathy and provided new ideas and strategies for further research and treatment of septic cardiomyopathy.

## Materials and Methods

### Experimental Materials and Instruments

The major experimental materials used in this study consisted of nicorandil (Jiangsu Hengrui Pharmaceuticals Co., Ltd.), biochemical kits (LDH, cTnI, CK-MB, GSH, MDA, total iron) (Nanjing Jiucheng Technology Co., Ltd.), Fe^2+^ assay kit (Abcam, Shanghai, China), normal saline (Guangzhou Shunfeng Technology Co., Ltd), chemiluminescent western blotting materials (Beijing Applygen Technologies Inc.), RIPA lysis buffer (Shanghai Bestbio Co., Ltd.), protein 26616 Marker (Thermo Fisher Scientific Inc., America), skimmed milk powder (Amresco, America), SYBR^™^ GreenER^™^ qPCR SuperMixes Universal (Thermo Fisher Scientific Inc., America), and protein antibodies (Proteintech North America (HQ) Proteintech Group, Inc.). Additionally, polyvinylidene fluoride (PVDF) membrane (Merck Millipore, Germany), 30% polyacrylamide, NaCl, tris base (trihydroxymethyl aminomethane), sodium dodecyl sulfate (SDS), glycine, tetramethylethylenediamine (TEMED), and absolute alcohol were all purchased from Guangzhou Shunfeng Biology Science and Technology Co., Ltd. The main experimental instruments included microplate reader (BioTek), UV-VIS Spectrophotometer (BioSpec-nano, Shimadzu), Leica RM2245 microtome, Leica optical microscope, FA2004 electronic analytical balance (Shanghai Yoke Instrument Co., Ltd.), and ASD-310S cryogenic centrifugal machine (Sigma).

### Animal Modeling and Grouping

Healthy SPF grade male SD rats (age: 5–6 weeks: weight: 180 ± 20 g) were provided by the Experimental Animal Center of People’s Hospital of Xinjiang Uygur Autonomous Region. The rats were adaptively bred for 14 days before the formal experiment. Next, the rats were randomly divided into 4 groups (*n* = 6): control group, nicorandil (Nic) group, LPS group, and LPS + Nic group. In the control group, the rats were fed normally and given the same amount of normal saline by gavage every day. In the nicorandil (Nic) group, the rats were fed normally and gavaged with nicorandil (15 mg/kg) [20] daily for 7 days. In the LPS group, the rats were intragastrically administered with the same amount of normal saline daily, and 7 days later, they were injected with LPS (10 mg/kg BW) intraperitoneally [22]. In the LPS + Nic group, the rats were gavaged with nicorandil (15 mg/kg) daily for 7 consecutive days, and on the 7th day, LPS was injected into the rats intraperitoneally. Heart tissue and serum were collected from rats 12 h after LPS injection. The ultrastructural features of myocardial tissue of rats were observed by transmission electron microscopy. This experiment was approved by the animal ethics committee of People’s Hospital of Xinjiang Uygur Autonomous Region (SYDW20231112207).

### Echocardiography

Rats were anesthetized with 2% isoflurane and kept warm on a heated platform. Then, the body temperature and electrocardiogram of the rats were continuously monitored. Next, cardiac function and morphology in diastole and systole were evaluated using a visualsonic Vevo 2100 high-resolution imaging system and a high-resolution (38 MHz) transducer. Specifically, the measured data included left ventricular internal dimensions in diastole (LVIDd; mm) and systole (LVIDs; mm), left ventricular posterior wall thicknesses in diastole (LVPWthd; mm) and systole (LVPWths; mm), and interventricular septum thickness in diastole (IVSthd [mm]) and systole (IVSths [mm]), and fractional shortening (FS, %). Based on these data, left ventricular ejection fraction (LVEF) (%), cardiac output (CO, mL/min), and stroke volume (SV, μL) were calculated [23].

### Biochemical Testing

The supernatant was collected from rat serum, and the homogenate supernatant was acquired from their myocardial tissue. Lactic dehydrogenase (LDH, A020-2-2), cardiac troponin I (cTnI, H149-2-2), and creatine kinase-MB (CK-MB, H197-1-2) in rat serum and GSH (A006-2-1), malondialdehyde (MDA, A003-1-2), total iron (A039-2-1), and Fe^2+^ (ab83366) in myocardial tissue were tested according to the kit instructions.

### H&E Staining

The animal tissues were sectioned, placed into a glass jar filled with xylene, and dewaxed twice (15 min/once). Next, the sections were dehydrated with absolute alcohol (5 min), 95% alcohol (5 min), 85% alcohol (5 min), and 75% alcohol (5 min) in turn. Subsequently, the samples were washed with running water for 10 min, stained with hematoxylin for 2 min, then washed again using running water for 10 min. The sections were blued by hydrochloric acid; seconds later, they were washed with running water for 10 min. After staining with eosin (70% alcohol) for 10 min, the sections were rinsed with absolute alcohol (5min/twice). Then, the samples were treated with xylene twice (5 min/once). After that, the sections were sealed with neutral resin and covered with slides. After dry, three or more different fields of view under a microscope were selected to observe the staining effect of tissues.

### qRT-PCR

Total RNA was extracted from cells in each group using TRIzol reagent, followed by reverse transcription into cDNA. The qRT-PCR system was configured using SYBR^™^ GreenER^™^ qPCR SuperMixes Universal and performed through StratageneMX3000P qPCR instrument according to the instructions. The cDNA amplification reaction system was shown as followed: initial denaturation at 95 °C for 10 min; denaturation at 95 °C for 15 s, for 40 cycles; annealing at 60 °C for 30 s; and extending at 72 °C for 30 s. Subsequently, Ct values of the target gene and internal reference gene were determined. Additionally, the 2^−ΔΔCt^ method was used to calculate the relative gene expression. The primer sequences were displayed as follows: TLR4: 5′-AGCCACGCATTCACAGGG-3′ and 5′-CATGGCTGGGATCAGAGTCC-3′, SLC7A11: 5′-TCTCCAAAGGAGGTTACCTGC-3′ and 5′-AGACTCCCCTCAGTAAAGTGAC-3′, and GAPDH: 5′-ATGGGGAAGGTGAAGGTCG-3′ and 5′-GAGGTCAATGAAGGGGTCAT- 3’.

### Western Blot

The total protein was extracted from cardiac myocytes of rats in each group and quantified. Then, the proteins were separated through sodium dodecyl sulfate-polyacrylamide gel electrophoresis (SDS-PAGE) and transferred to PVDF membranes. Next, the membranes were sealed with 5% skimmed milk at ambient temperature for 1 h. Subsequently, the primary antibody glutathione peroxidase 4 (GPX4, 67763–1-Ig), SLC7A11 (26864–1-AP), ACSL4 (66617–1-Ig), DMT1 (20507–1-AP), TfR1 (10084–2-AP), Fpn (26601–1-AP), and GAPDH (10494–1-AP) were diluted to 1:1000 and incubated with the membranes overnight at 4 °C. Next day, the membranes were washed and incubated with secondary antibodies at ambient temperature. The dilution ratio of goat anti-rat antibodies (ZB-2305) and goat anti-rabbit IgG secondary antibody (ZB2301) was 1:10000. After 1 h, the membranes were washed again. The developer was prepared using ultra-sensitive enhanced chemiluminescent (ECL) in the light of the production instruction. After exposure, the images of proteins were obtained and the relative protein expression levels were analyzed using the ImageJ software with GAPDH as the internal control.

### Cell Culture

Cardiomyocyte H9C2 cells (CRL-1446) were purchased from American Type Culture Collection (ATCC) and cultured at Dulbecco’s modified Eagle’s medium (DMEM) admixed with 10% fetal bovine serum (FBS) and 1% penicillin-streptomycin (Sigma-Aldrich, Castle Hill, NSW, Australia). Then, they were placed within a 5% CO_2_ incubator at 37 °C. Notably, the medium was replenished every 4 days and the cells were not passaged until 95% confluence. Cells from passages 3 to 5 were used for further experiment.

### Cell Transfection

H9C2 cells were inoculated in six-well plates at 40% confluence before transfection. The negative control (NC, forward: 5′-GCCCGUAGAACACGUCGUAdTdT-3′, reverse: 5′-UACGACGUGUUCUACGGGCdTdT-3′), TLR4 siRNA (forward: 5′-GGAUCUUUCUUAUAACUAUdTdT-3′, reverse: 5′-AUAGUUAUAAGAAAGAUCCdTdT-3′) were designed and synthesized by Invitrogen (Camarillo, CA, USA). cDNA encoding human SLC7A11 was cloned into pcDNA3.1 vector and purified using Maxiprep (Promega). The H9C2 cells were transfected with lipofectamine 2000 (Invitrogen, Camarillo, CA, USA) for 48 h following the manufacturer’s instructions. After transfection, the lipofectamine solution was removed, and the DMEM containing FBS was added.

### Cell Grouping

H9C2 cells were cultured at 37 °C and divided into 12 groups. Specifically, H9C2 cells in the siNC group and si-TLR4 group were transfected with negative siRNA and TLR4 siRNA, respectively. H9C2 cells in the vector group and SLC7A11-OE group were transfected with blank vector and SLC7A11 pcDNA3.1 vector, respectively. In the LPS group, H9C2 cells were treated with LPS (0.5 μg/mL). Before LPS treatment, cells in the LPS + Nic, LPS + siNC, LPS + si-TLR4, LPS + vector, and LPS + SLC7A11-OE groups were treated with nicorandil (100 μM) or combined with transfected with negative siRNA, transfected with TLR4 siRNA, transfected with blank vector, and transfected with SLC7A11 pcDNA3.1 vector, respectively. As for cells in the LPS + Nic + si-TLR4 and LPS + Nic + SLC7A11-OE groups, they were transfected with TLR4 siRNA or SLC7A11 pcDNA3.1 vector, respectively, then treated with nicorandil and induced with LPS.

### MTT Examination

Cell viability was assessed using the 3-(4,5-dimethylthiazol-2-yl) − 2,5-diphenyltetrazolium bromide (MTT) assay. To be specific, 10 mL (10% v/v) of MTT (5 mg/mL MTT was mixed with PBS) was added into each well. Next, the cells were further incubated for 4 h at 37 °C. After removal of the complete culture medium, the cultured cells were supplemented with 100 μL isopropyl alcohol, and the optical density was measured at 570 nm.

### Statistical Analysis

All experimental data were statistically analyzed using the SPSS10. 0 software. *T*-test was used for comparison between two groups, and one-way analysis of variance for comparison among multiple groups. The results were expressed as mean ± standard deviation (SD), and *P* < 0.05 indicated significantly different.

## Results

### Nicorandil Improves Cardiac Function of Rats with Septic Cardiomyopathy

**Fig. 1:**
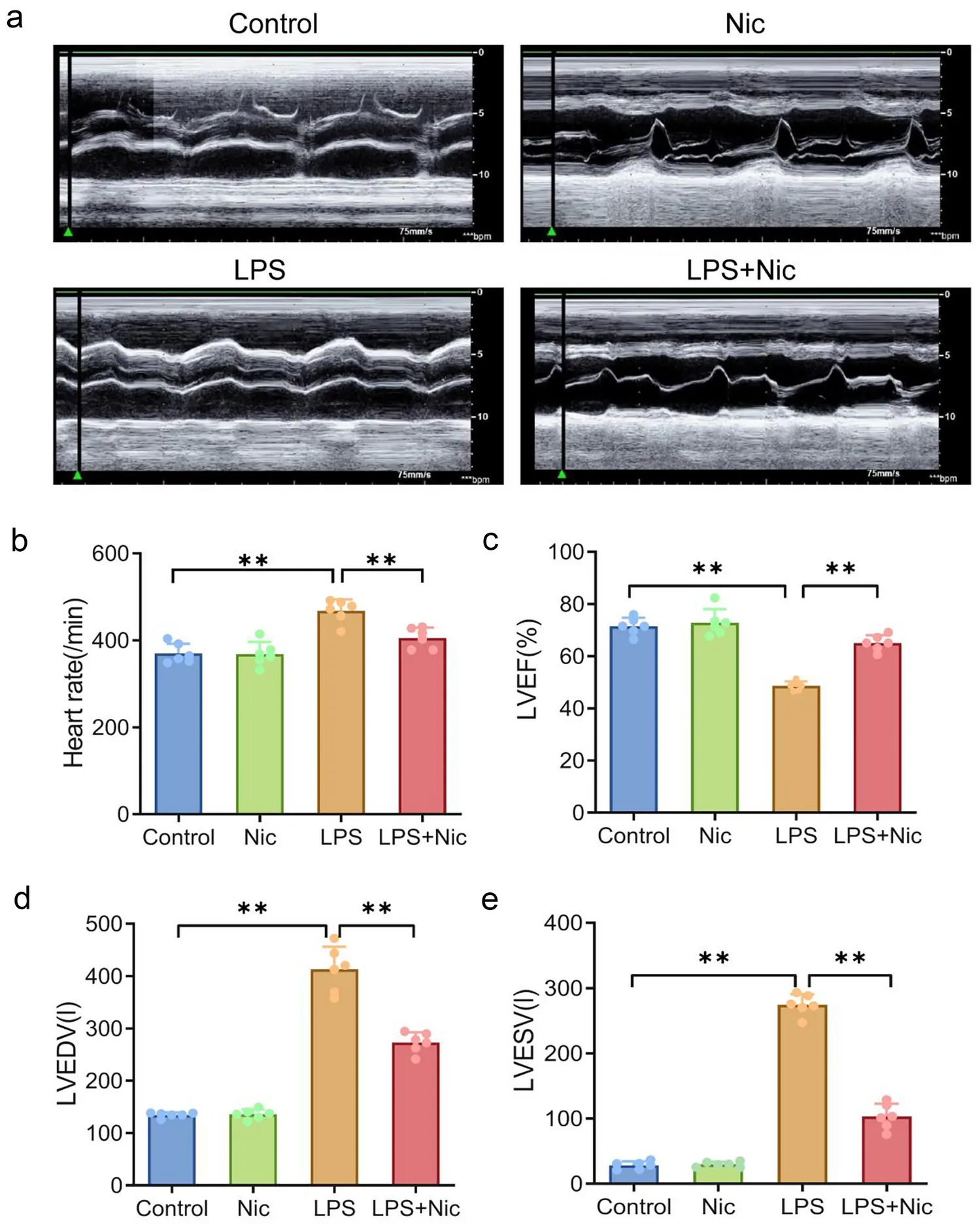
The improving effect of nicorandil on cardiac function of rats with septic cardiomyopathy. **a** Representative images of echocardiography in the control, Nic, LPS, and LPS + Nic groups. The heart rate (**b**), LVEF (**c**), LVEDV (**d**), and LVESV (**e**) of rats in the control, Nic, LPS, and LPS + Nic groups. **P < 0.01. LVEF, left ventricular ejection fraction; LVEDV, left ventricular end-diastolic volume; LVESV, left ventricular end-systolic volume.

The results of echocardiography showed that the heart rate of rats in LPS group were significantly higher than the control and Nic groups, while Nic significantly reduce the heart rate of LPS rats (Fig. 1a, b). Besides, compared with the control group, LVEF was significantly decreased while left ventricular end-diastolic volume (LVEDV) and left ventricular end-systolic volume (LVESV) were significantly increased in the LPS group. Relative to the LPS group, the LPS + Nic group exhibited notably raised LVEF and markedly declined LVEDV and LVESV. As for the control group and the Nic group, the differences between them were not statistically significant (Fig. 1c–e). Collectively, nicorandil could improve the cardiac function damage in rats with LPS-induced septic cardiomyopathy.

### Nicorandil Attenuates Myocardial Injury in Rats with Septic Cardiomyopathy

**Fig. 2:**
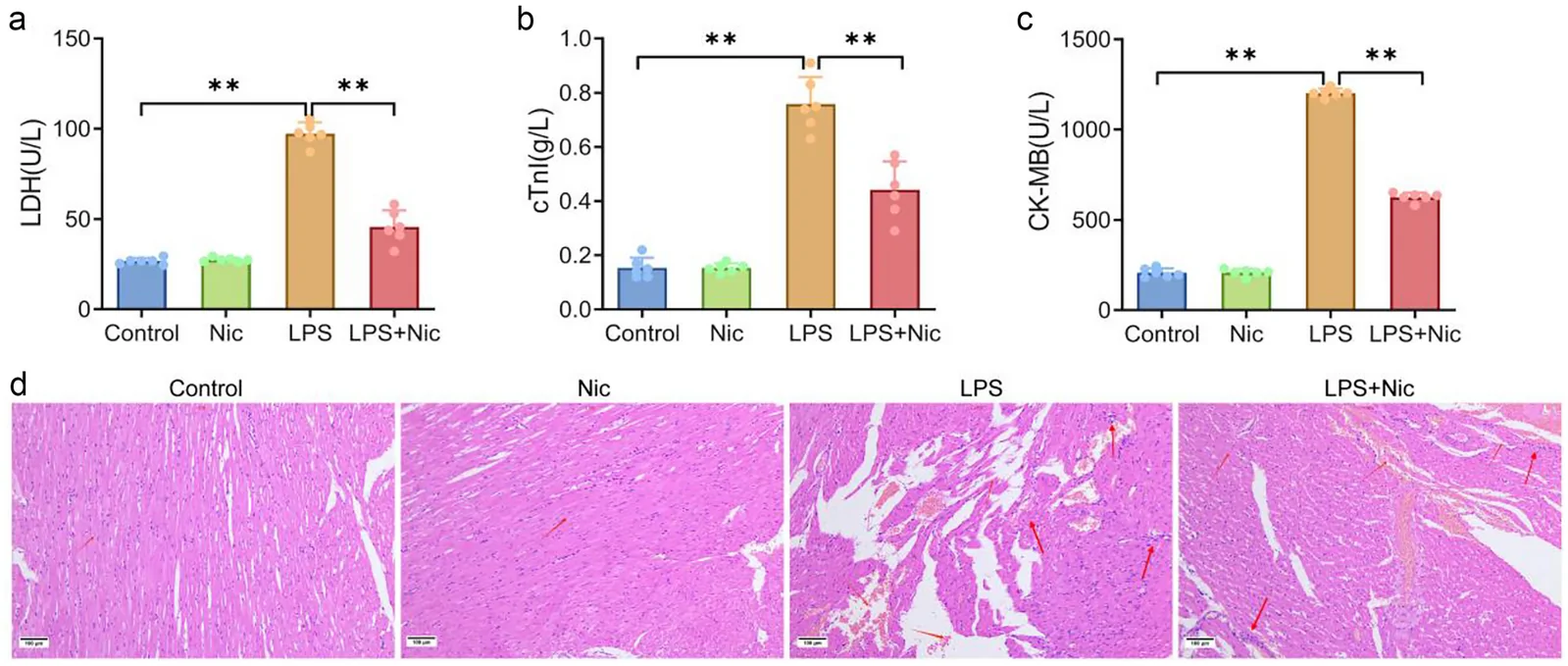
Nicorandil attenuates myocardial injury in rats with septic cardiomyopathy. Biochemical test was performed to determine the levels of LDH (**a**), cTnI (**b**), and CK-MB (**c**) in rat serum in the control, Nic, LPS, and LPS + Nic groups. **d** H&E staining of myocardial tissue in the control group, Nic group, LPS group, and LPS + Nic group. The red arrows indicated pathological changes. Scale bar = 100 µm. **P < 0.01. LDH, lactic dehydrogenase; cTnI, cardiac troponin I; CK-MB, creatine kinase-MB.

Serum biochemical test results displayed that compared with the control group, the serum LDH, cTnI, and CK-MB levels in the LPS group were significantly increased; in contrast to the LPS group, the levels of serum LDH, cTnI, and CK-MB in the LPS + Nic group were much lower. Although the levels of LDH, cTnI, and CK-MB in the Nic group were slightly lower than those in the control group, the differences were not statistically significant (Fig. 2a–c). Besides, according to the outcomes of HE staining, samples in the control group and the Nic group presented normal myocardial structure and no histological changes. However, the LPS group samples showed increased eosinophils, irregular arrangement of cardiac myocytes, karyolysis, and fragmentation. All in all, nicorandil treatment could improve the histological structure of the myocardium with mild degeneration (Fig. 2d).

### Nicorandil Relieves Ferroptosis in Myocardial Tissues of Rats with Septic Cardiomyopathy

**Fig. 3:**
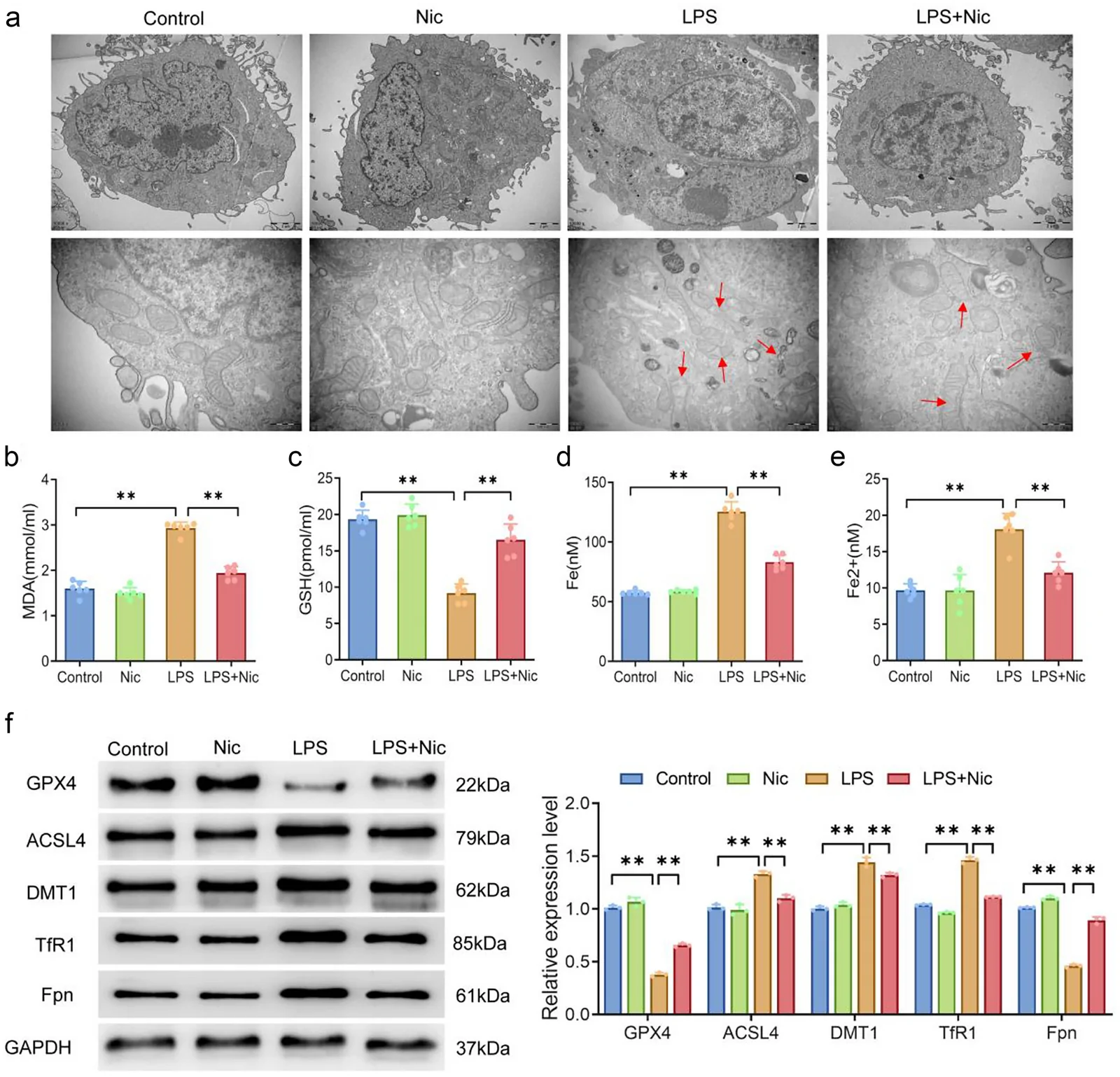
Nicorandil attenuates ferroptosis in myocardial tissue of rats with septic cardiomyopathy. **a** The ultrastructural features of myocardial tissue of rats were observed by transmission electron microscopy. The red arrow manifest mitochondrial membrane rupture, mitochondrial atrophy, and the decrease or disappearance of mitochondrial cristae (scale bar = up: 2 μm/down: 500 nm). Biochemical test was applied to determine the levels of MDA (**b**), GSH (**c**), total iron (**d**), and Fe^2+^ (**e**) in myocardial tissues in the control group, Nic group, LPS group, and LPS + Nic group. **f** The protein levels of GPX4, ACSL4, and DMT1 in the myocardial tissues of rats in the control group, Nic group, LPS group, and LPS + Nic group were detected by western blot. **P < 0.01. MDA, malondialdehyde; GSH, glutathione.

To explore the effect of Nic on ferroptosis, changes in the mitochondrial ultrastructure in the rat myocardium were observed by transmission electron microscopy. The results showed that no significant change in myocardial mitochondrial morphology was seen in the control and Nic groups, whereas the cells in the LPS group showed obvious rupture of the mitochondrial membrane, mitochondrial atrophy, and reduction or disappearance of the mitochondrial cristae and these conditions were significantly improved after Nic treatment (Fig. 3a). Additionally, relative to the control group, the levels of MDA, total iron, and Fe^2+^ were notably increased, while the GSH level was markedly decreased in the myocardial tissue of rats in the LPS group. In comparison with the LPS group, the levels of MDA, total iron, and Fe^2+^ in the myocardial tissue of rats in the LPS + Nic group were remarkably reduced, and the GSH level was markedly increased (Fig. 3b–e). The findings of western blot revealed that relative to the control group, the protein levels of GPX4 and Fpn were clearly reduced, while the protein levels of ACSL4, DMT1, and TfR1 were significantly increased in the myocardial tissue of rats in the LPS group. Moreover, compared with the LPS group, the protein levels of GPX4 and Fpn were notably increased, while the protein levels of ACSL4, DMT1, and TfR1 were observably decreased in the myocardial tissues of the LPS + Nic group (Fig. 3f). In a nutshell, nicorandil could reduce ferroptosis in the myocardial tissues of rats with septic cardiomyopathy.

### Nicorandil Improves Septic Cardiomyopathy Through Toll-Like Receptor (TLR) 4/Solute Carrier (SLC) 7A11 Signaling Pathway

**Fig. 4:**
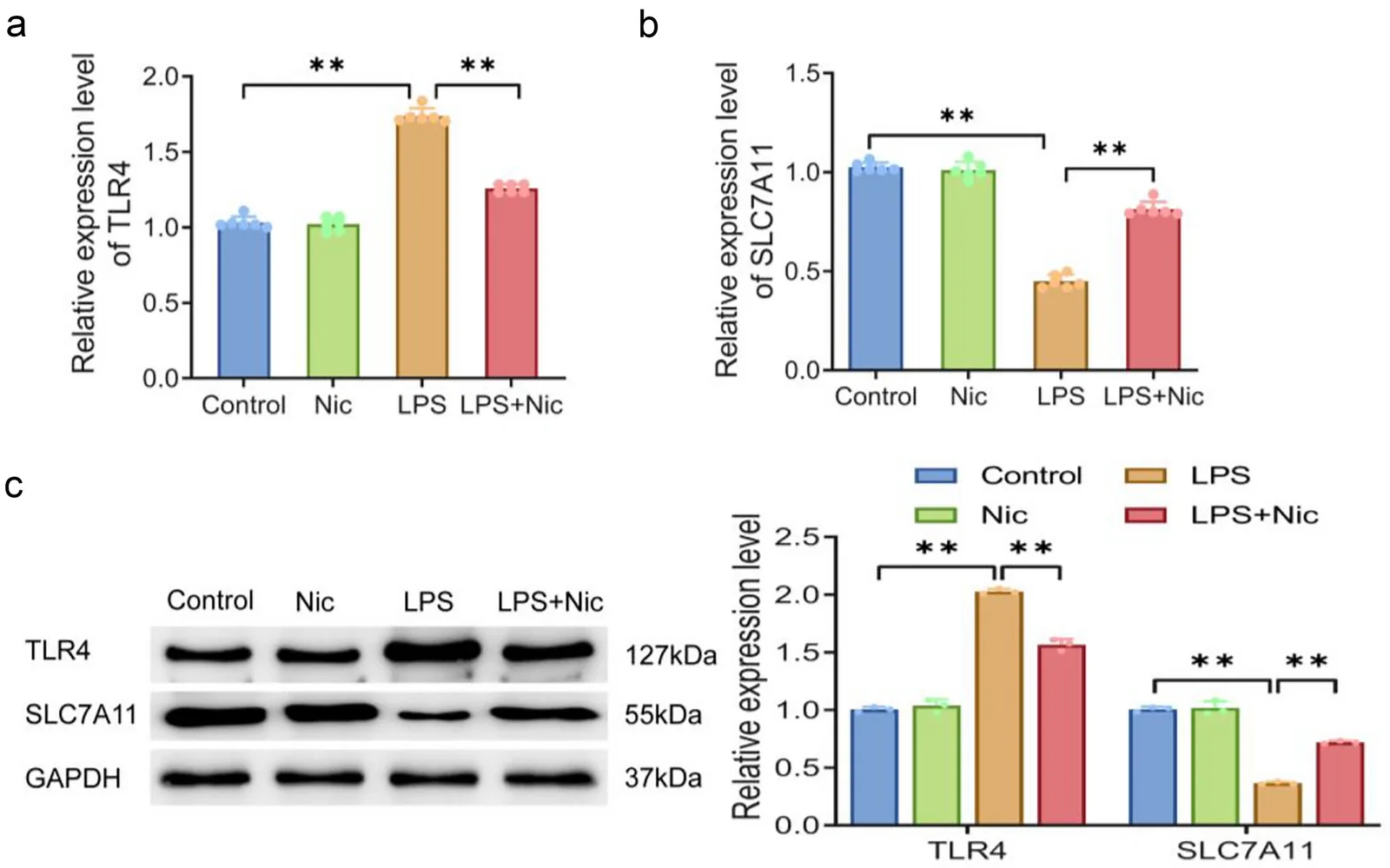
Effects of nicorandil on Toll-like receptor (TLR) 4/solute carrier (SLC) 7A11 signaling pathway in rats with septic cardiomyopathy. The levels of TLR4 (**a**) and SLC7A11 (**b**) in myocardial tissues of rats in the control group, Nic group, LPS group, and LPS + Nic group detected by qRT-PCR. **c** The protein levels of TLR4 and SLC7A11 in myocardial tissues of rats in the control group, Nic group, LPS group, and LPS + Nic group measured by western blot. **P < 0.01. TLR, toll-like receptor; SLC, solute carrier.

The results of qRT-PCR exhibited that compared with the control group, the expression level of TLR4 was notably up-regulated, while the level of SLC7A11 was markedly downregulated in the myocardial tissues of rats in the LPS group. As opposed to the LPS group, the LPS + Nic group exhibited notably decreased expression level of TLR4 while obviously increased level of SLC7A11 in the myocardial tissues (Fig. 4a, b). Besides, the results of western blot also confirmed that nicorandil could reduce the protein level of TLR4 while increasing the protein level of SLC7A11 in the tissues (Fig. 4c). Briefly speaking, nicorandil could improve septic cardiomyopathy through the TLR4/SLC7A11 signaling pathway.

### Knockdown of TLR4 Strengthens the Inhibition Effect of Nicorandil on Ferroptosis in LPS-Induced H9C2 Cells

**Fig. 5:**
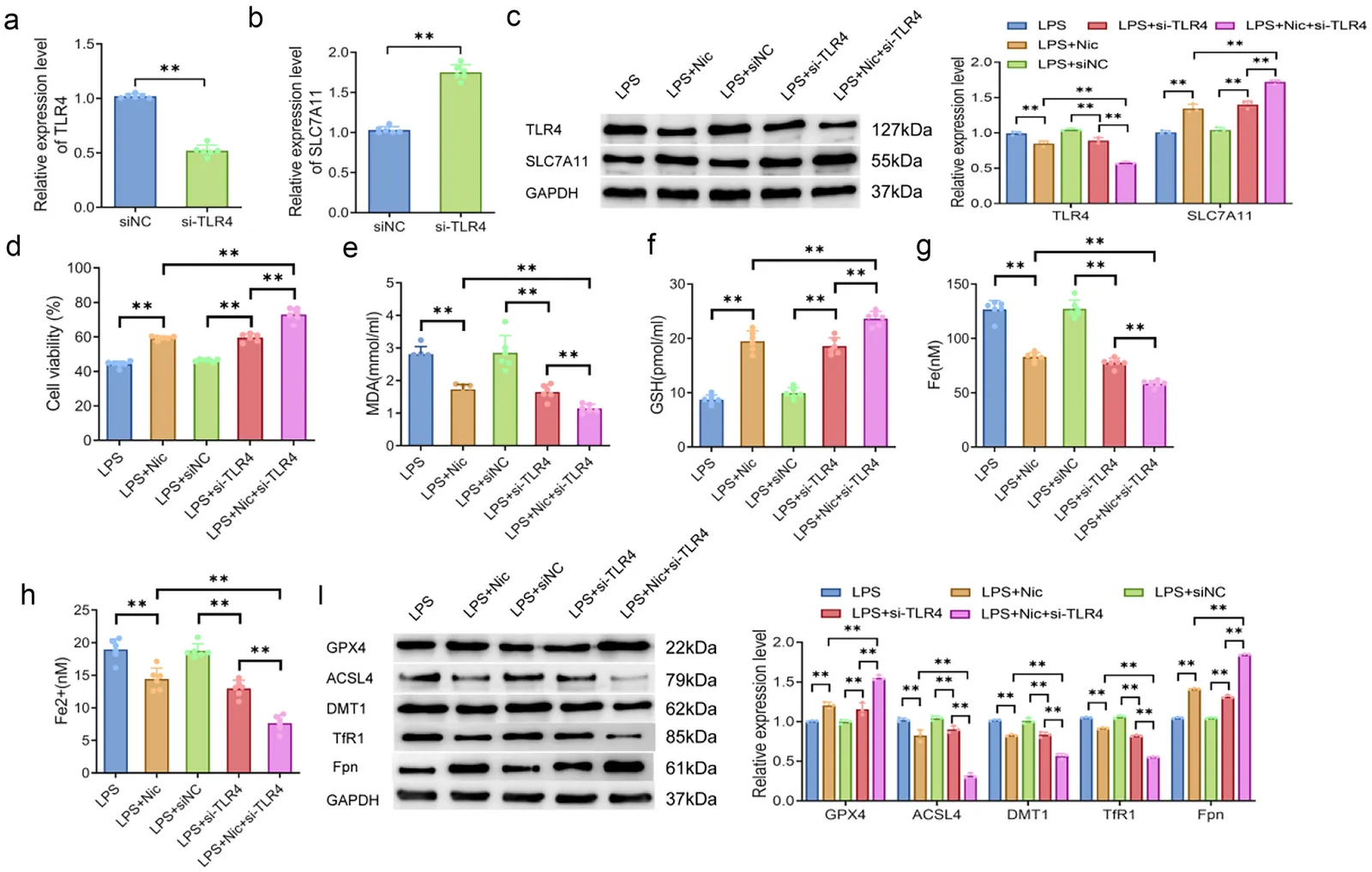
Knockdown of TLR4 strengthens the inhibition effect of nicorandil on ferroptosis in LPS-induced H9C2 cells. **a**–**c** qRT-PCR and Western blot to measure the expression of TLR4 and SLC7A11. **d** MTT to measure the cell proliferation levels of each group. **e**–**h** The levels of MDA, GSH, total iron, and Fe^2+^ were detected biochemically. **i** Western blot to measure GPX4, ACSL4, DMT1, TfR1, and Fpn protein levels in cells of each group. **P < 0.01. **P < 0.01.

As shown in Fig. 5, the si-TLR4 group displayed an obvious drop in expression level of TLR4 and a marked rise in expression level of SLC7A11 (Fig. 5a–c). The outcomes of MTT included that the cell proliferation level was notably increased in the LPS + si-TLR4 group and LPS + Nic + si-TLR4 groups in comparison with the LPS + siNC group. Similarly, relative to the LPS + Nic group, the proliferation level of cells in the LPS + Nic + si-TLR4 group was obviously increased (Fig. 5d). In addition, the levels of MDA, total iron, and Fe^2+^ were much lower and the GSH level was much higher in the LPS + si-TLR4 group than those in the LPS + siNC group. In contrast to the LPS + Nic group, the LPS + Nic + si-TLR4 group displayed notably reduced levels of MDA, total iron, and Fe^2+^, as well as significantly increased GSH level (Fig. 5e–h). In regard to ferroptosis-related protein detection results, relative to the LPS + siNC group, the protein level of GPX4 and Fpn was markedly increased, while the protein levels of ACSL4, DMT1, and TfR1were notably decreased in the LPS + si-TLR4 group. Additionally, the LPS + Nic + si-TLR4 group exhibited much higher protein levels of GPX4 and Fpn while notably lower protein levels of ACSL4, DMT1, and TfR1 than the LPS + Nic group (Fig. 5i). The above findings indicated that knocking down TLR4 expression could further promote the effect of nicorandil on promoting cell proliferation and inhibiting ferroptosis.

### Overexpression of SLC7A11 Aids in the Inhibition of Ferroptosis by Nicorandil in LPS-Induced H9C2 Cells

**Fig. 6:**
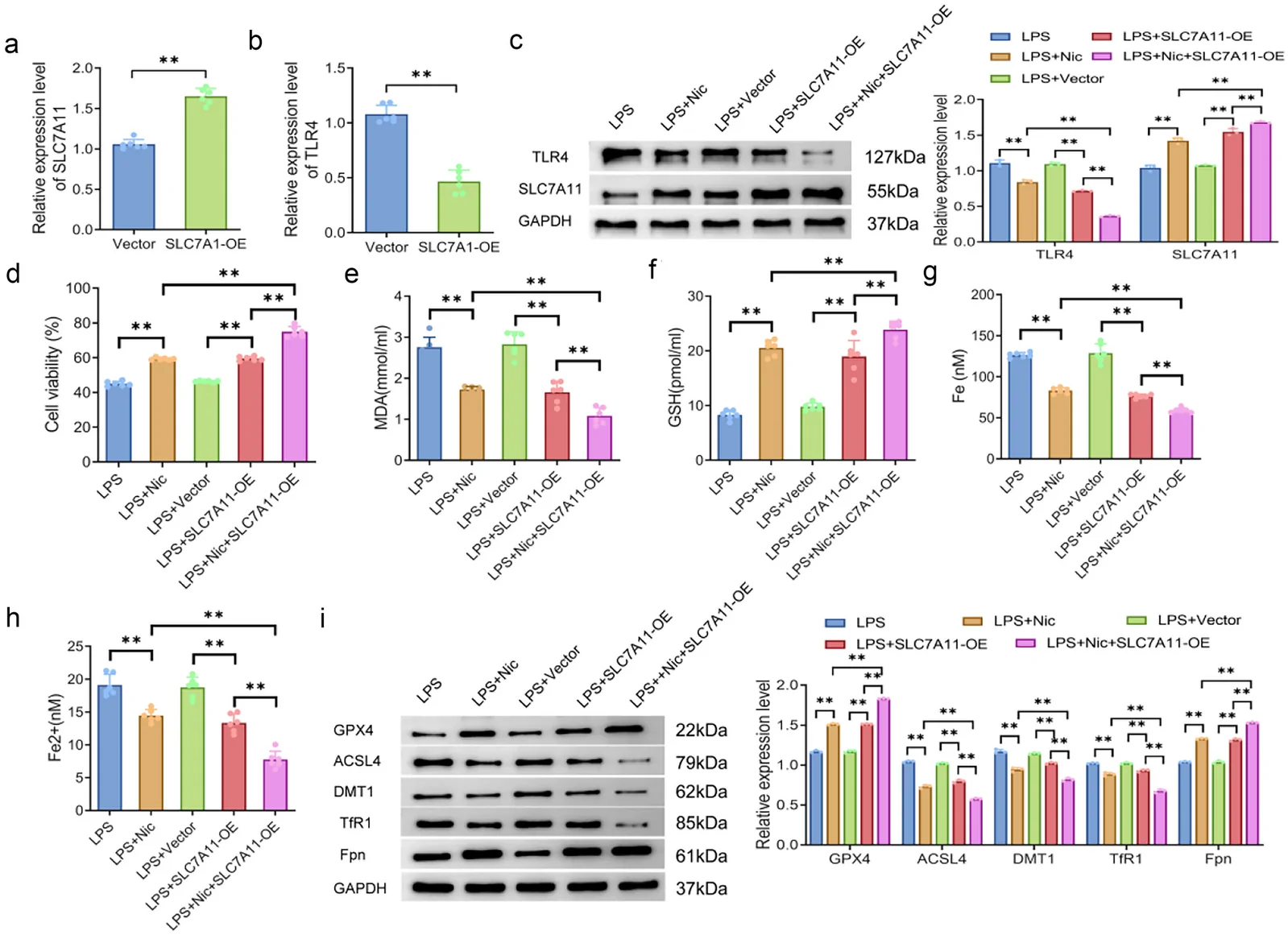
Overexpression of SLC7A11 aids in the inhibition of ferroptosis by nicorandil in LPS-induced H9C2 cells. **a**–**c** qRT-PCR and Western blot to measure the expression of SLC7A11 and TLR4, **d** MTT to measure the cell proliferation levels of each group. **e**–**h** The levels of MDA, GSH, total iron, and Fe^2+^ were detected biochemically. **i** Western blot to measure GPX4, ACSL4, DMT1, TfR1, and Fpn protein levels in cells of each group. **P < 0.01.

As shown in Fig. 6, the SLC7A11-OE group displayed a remarked rise in SLC7A11 level and a decline in TLR4 level (Fig. 6a–c). The MTT results revealed a much higher cell proliferation level in the LPS + SLC7A11-OE group and LPS + Nic + SLC7A11-OE group than in the LPS + vector group. Similarly, relative to the LPS + Nic group, the proliferation level of cells in the LPS + Nic + SLC7A11-OE group was obviously increased (Fig. 6d). Furthermore, compared with the LPS + vector group, the levels of MDA, total iron, and Fe^2+^ were notably reduced, while the GSH level was obviously upregulated in the LPS + SLC7A11-OE group. In contrast to the LPS + Nic group, a notable decline in levels of MDA, total iron, and Fe^2+^ as well as an obvious rise in GSH level were observed in the LPS + Nic + SLC7A11-OE group (Fig. 6f–h). As for ferroptosis-related protein detection results, the protein level of GPX4 and Fpn was found to be markedly increased, while the protein levels of ACSL4, DMT1, and TfR1 to be notably decreased in the LPS + SLC7A11-OE group relative to the LPS + vector group. And the LPS + Nic + SLC7A11-OE group exhibited much higher protein levels of GPX4 and Fpn but much lower protein levels of ACSL4, DMT1, and TfR1 than the LPS + Nic group (Fig. 6i). It was evident that overexpression of SLC7A11 could further promote the effect of nicorandil on promoting cell proliferation and inhibiting ferroptosis.

## Discussion

Sepsis is a systemic inflammatory response syndrome with yearly rising rates of morbidity and mortality. The heart is an important target organ for pathophysiological process of sepsis-induced organ failure. Previous studies have presented a significant increase in ICU admissions, mean hospital stay, and mortality in patients with septic cardiomyopathy [24]. Early diagnosis and effective treatment are critical for improving clinical prognosis of patients with septic cardiomyopathy. In recent years, the popularity of examinations such as bedside echocardiography has deepened people’s understanding to septic cardiomyopathy. However, the pathogenesis of septic cardiomyopathy has not been fully elucidated. Currently, septic cardiomyopathy is believed a link in the development of septic multiple organ dysfunction. Systemic inflammatory response not only directly damages myocardium but also induces cardiac dysfunction by affecting coronary artery microcirculation of heart, mitochondrial function of cardiac myocytes, adrenergic receptor of heart, calcium ion transport of cardiac myocytes, and cardiac myocyte apoptosis [25, 26]. Therefore, early evaluation, dynamic monitoring, and timely intervention are essential for the protection of the cardiac function and the improvement of the survival and prognosis of patients with septic cardiomyopathy.

In this study, the results of echocardiography showed that nicorandil could significantly increase the LVEF of rats while notably reducing the LVEDV and LVESV. Therefore, nicorandil could be applied to treat rats with septic cardiomyopathy. Xing *et al*. [27] also discovered through animal research that nicorandil improved cardiac function of rats with acute heart failure and that after long-term use, it reduced left ventricular hypertrophy and fibrosis and effectively delayed ventricular remodeling. The mechanism of nicorandil may be related to its function in inhibiting left ventricular myocardial cell apoptosis–related gene expression. LDH, cTnI, and CK-MB are important myocardial injury markers with high sensitivity and specificity [28], which are of great significance for the diagnosis of myocardial infarction and risk stratification. Studies have claimed that patients with septic cardiomyopathy are prone to myocardial injury, which is usually accompanied by increased levels of serum LDH, cTnI, and CK-MB. Notably, the increased LDH, cTnI, and CK-MB expression levels, reflecting the severity of myocardial cell injury, are closely associated with the severity of the disease and mortality [29, 30]. In this paper, the levels of the three indicators were above average, while after treatment, they returned to normal levels. Similar to the findings of the study by Kawakita *et al*. [31], this study also suggested that in addition to reducing the expression of myocardial injury biomarkers, nicorandil may partially reverse myocardial injury by enhancing cardiac function and improving myocardial blood supply.

Ferroptosis is an iron-dependent type of non-apoptotic cell death caused by lipid peroxidation [32]. Recently, with the in-depth study on the role of ferroptosis in the heart, there is increasing evidence that ferroptosis has a bearing on the occurrence of septic cardiomyopathy [33, 34]. Generally, ferroptosis is induced by abnormal iron metabolism and excessive lipid peroxidation accumulation. For iron metabolism pathway, transferrin with Fe^3+^ enters intracellular cells *via* TfR1, and Fe^3+^ is converted into Fe^2+^ by STEAP3; then, Fe^2+^ is released into the cytoplasm by DMT1. The Fenton reaction, which involves Fe^2+^, generates L-ROS and encourages ferroptosis. The excess of Fe^2+^ was transferred out of the cell by ferroportin [35]. For lipid metabolism pathway, polyunsaturated fatty acids (PUFAs) derived from lipid bilayers combined with phosphatidylethanolamine (PE) are metabolized to PUFA-PE by ACSL 4 and LPCAT, and then oxidized by lipoxygenase (LOX) to generate large amounts of ROS (L-ROS) to induce ferroptosis [36]. It is worth of noting that GPX4 makes the peroxygenic bond (L-OOH) loses its peroxide activity by converting it into hydroxyl (L-OH) *via* GSH. This process resists the ferroptosis of cells [37, 38]. In this research, nicorandil could effectively reduce the levels of MDA, total iron, and Fe^2+^ in the myocardial tissues of rats and increase the level of GSH. Also, the results of western blot revealed notably raised protein level of GPX4 and Fpn while markedly declined protein levels of ACSL4, DMT1, and TfR1 in the myocardial tissues in the LPS + Nic group. In other word, nicorandil can significantly reduce ferroptosis in myocardial cells and improve cardiac function by inhibiting iron overload and lipid peroxidation in rats.

TLRs are activated by cellular patterns, including damage-associated molecular patterns (DAMPs). The main TLRs expressed in myocardial cells include TLR2, TLR3, and TLR410, wherein TLR4 is involved in myocardial injury caused by myocardial infarction. TLR4 is the most widely studied TLRs. TLR4 is activated in ischemic heart disease tissue. Not only does it promote the up-regulation of pro-inflammatory factors but also it brings additional damage to damaged myocardium. Myocardial inflammation can be reduced and heart function enhanced by inhibiting the TLR4 pathway. For example, suppressing the expression of TLR4 can reduce the expression of nlrp3-mediated inflammation and pro-inflammatory cytokines, the size of myocardial infarction, and myocardial tissue remodeling, as well as protect cardiac function [39, 40]. The SLC7A11 gene, a member of the solute transport family, is crucial for controlling ferroptosis [41]. Briefly, the SLC7A11 gene mediates the conversion of cystine to cysteine and inhibits the lipid peroxidation of PUFAs with high expression on cell membrane, thereby preventing cell ferroptosis [42]. In this paper, nicorandil reduced the LPS-induced increase in the protein level of TLR4 and significantly increased the protein level of SLC7A11. Moreover, knocking down TLR4 expression increased the expression of SLC7A11, while overexpression of SLC7A11 inhibited the expression of TLR4, all of which further strengthened the inhibitory effect of nicorandil on ferroptosis.

However, this study is limited to the exploration of TLR4/SLC7A11 signaling pathway, which is just one of many signaling pathways related to ferroptosis. Hence, the experiments need to be further improved in the future research by addressing this issue. Besides, due to possible interactions between the various signaling pathways, the study will be more convincing if rats after knocking down or silencing GPX4 and SLC7A11 genes can be involved.

## Conclusion

**Fig. 7:**
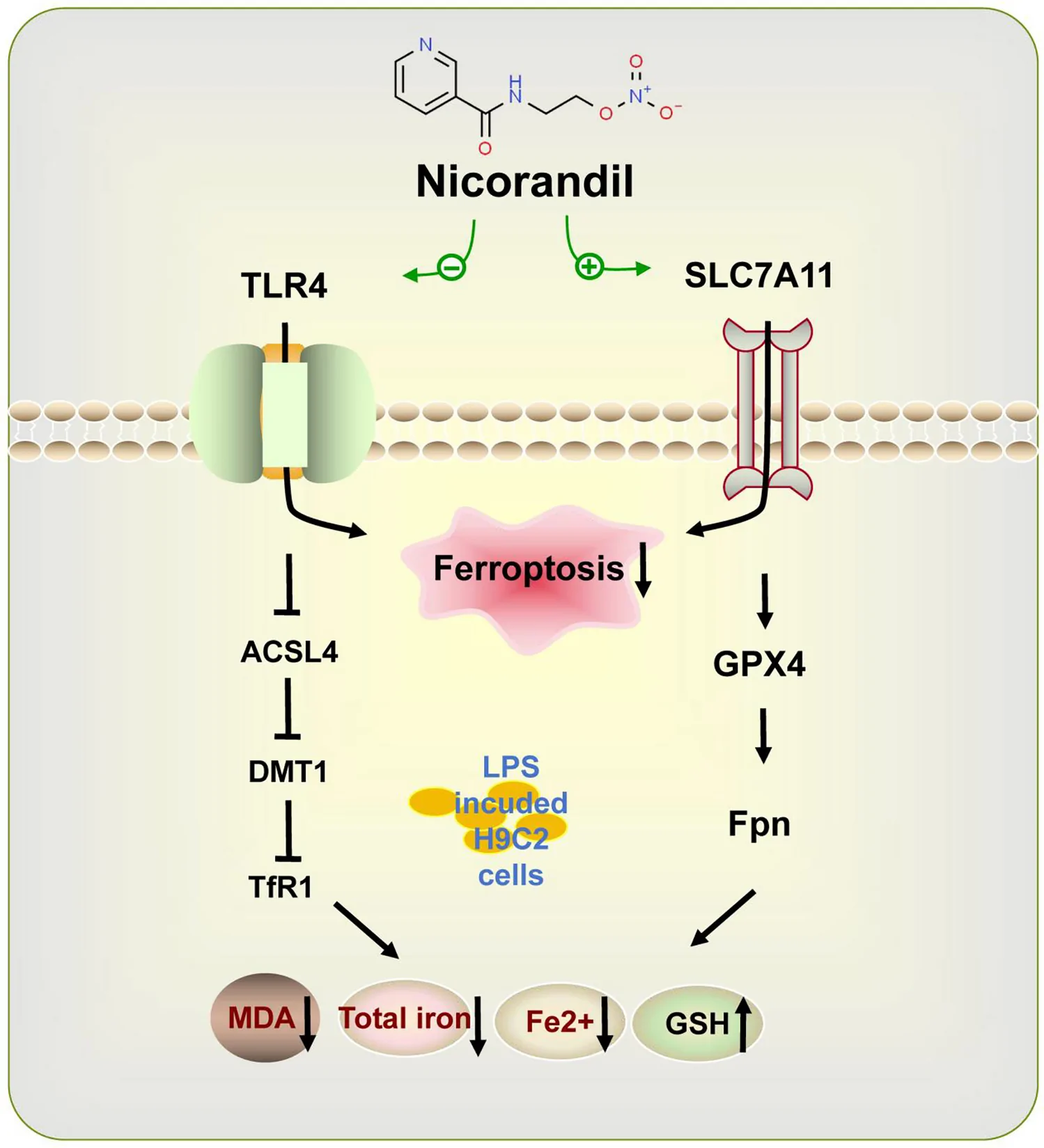
A mechanism diagram of the effect of nicorandil on ferroptosis.

To sum up, nicorandil can effectively treat the myocardial injury due to sepsis, and its mechanism may be achieved through inhibiting oxidative stress and activating TLR4/SLC7A11 signaling pathway (Fig. 7). All in all, nicorandil will be a new perspective and new strategy for the treatment of sepsis-induced myocardial injury.

## Data Availability

The data supporting the findings in this study will be made available upon reasonable request to the corresponding authors.
