# Correction to: Nicorandil Regulates Ferroptosis and Mitigates Septic Cardiomyopathy via TLR4/SLC7A11 Signaling Pathway

**DOI:** 10.1007/s10753-026-02625-0

**Published:** 2026-10-14

**Authors:** Jin-shuai Lu, Jian-hao Wang, Kun Han, Nan Li

**Affiliations:** 1 Departments of Emergency, People’s Hospital of Xinjiang Uygur Autonomous Region, Urumqi City, 830001 Xinjiang China; 2 Postgraduate School, Xinjiang Medical University, Urumqi City, 830017 Xinjiang China; 3 Xinjiang Emergency Center, People’s Hospital of Xinjiang Uygur Autonomous Region, Urumqi City, 830001 Xinjiang China

**Correction: Inflammation (2023) 47:975-988**

**https://doi.org/10.1007/s10753-023-01954-8**

The originally published version of this article contained mistakes in Figure 3.

The correct Figure 3 is displayed on this correction notice. 

**Figure:**
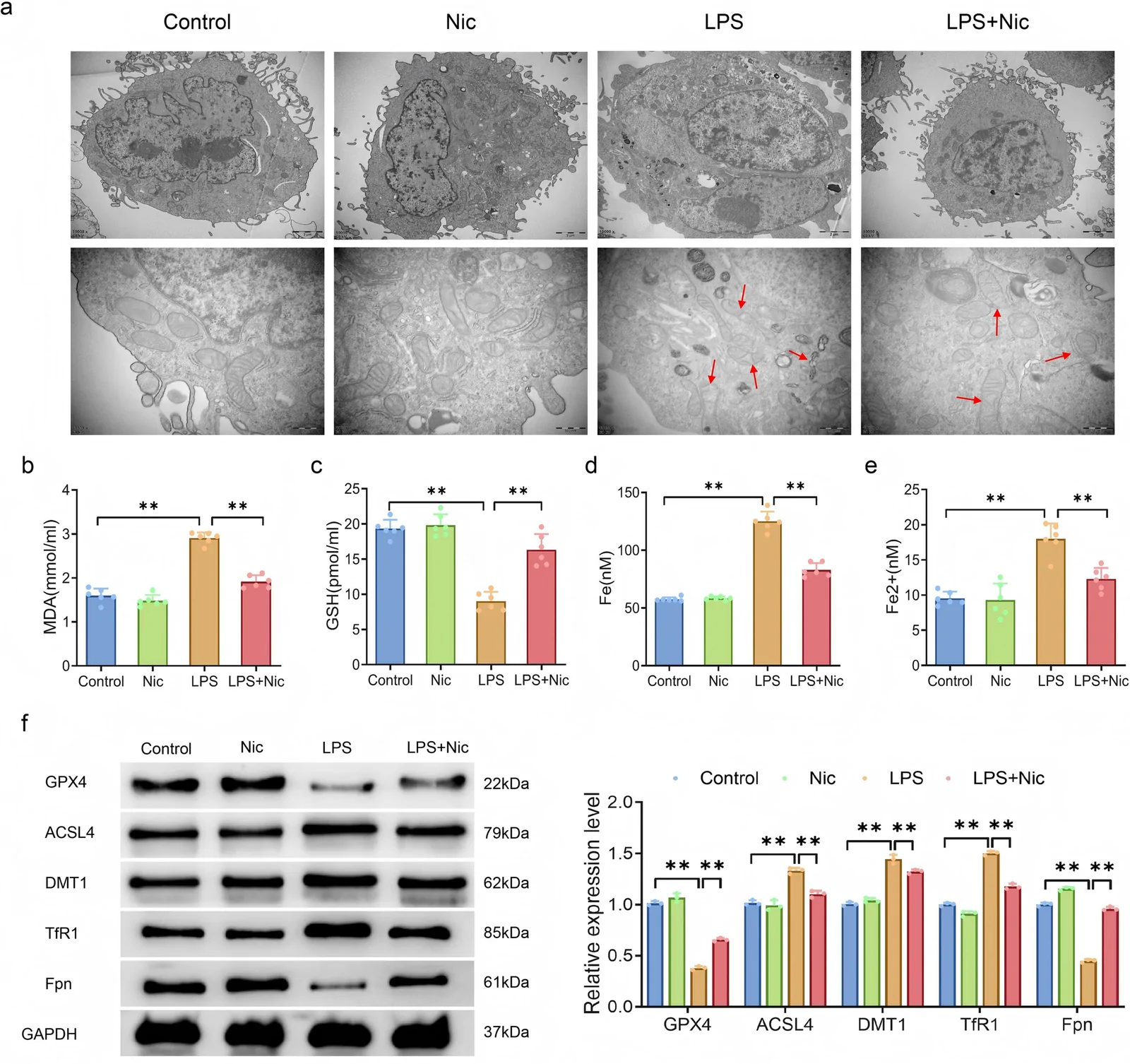

The authors apologizes for this oversight.

